# Temporal Activity Patterns of the Eurasian Beaver and Coexisting Species in a Mediterranean Ecosystem

**DOI:** 10.3390/ani12151961

**Published:** 2022-08-02

**Authors:** Emiliano Mori, Giuseppe Mazza, Chiara Pucci, Davide Senserini, Roisin Campbell-Palmer, Marco Contaldo, Andrea Viviano

**Affiliations:** 1 Consiglio Nazionale delle Ricerche, Istituto di Ricerca sugli Ecosistemi Terrestri, Via Madonna del Piano 10, 50019 Firenze, Italy; a.viviano@studenti.unipi.it; 2 CREA Research Centre for Plant Protection and Certification (CREA-DC), Via Lanciola 12/a, 50125 Firenze, Italy; giuseppe.mazza@crea.gov.it; 3 Independent Researcher, Str. di Pilli 1, 53035 Siena, Italy; chiara.pucci80@gmail.com; 4 Independent Researcher, Loc. Defizio, 58036 Grosseto, Italy; davidesenserini@gmail.com; 5 Beaver Trust, 61 Bridge Street, Kington HR5 3DJ, UK; roisin@beavertrust.org; 6 Independent Researcher, 53100 Siena, Italy; contald@yahoo.it

**Keywords:** activity rhythms, *C* *astor fiber*, *Canis lupus*, *Myocastor coypus*, moonlight avoidance, riverine ecosystems, Central Italy

## Abstract

**Simple Summary:**

Competition and predation are the main factors shaping interspecific coexistence in wildlife communities. Species reappearance and introductions may alter these dynamics. The Eurasian beaver *Castor fiber* is a widespread species in Europe, and it is part of the diet of many birds and mammalian carnivores of all sizes. Additionally, competition with other herbivorous mammals at feeding sites could occur. For this reason, we computed the overlap of activity rhythms among the recently reappeared Eurasian beaver and its potential predators (red fox *Vulpes vulpes* and grey wolf *Canis lupus*) and competitors (coypu *Myocastor coypus*) in Central Italy. The beaver was confirmed as a mostly crepuscular species, avoiding the bright moonlight nights and the activity of its predators, which is potentially shaped by the behaviour of their main prey.

**Abstract:**

Analyses of temporal partitioning and overlaps in activity rhythms are pivotal to shed light on interspecific coexistence between similar species or prey and predators. In this work, we assessed the overlap of activity rhythms between the Eurasian beaver *Castor fiber* and its potential competitors and predators through camera trapping in an area in Central Italy. Interspecific overlaps of temporal activity patterns were estimated for the beavers, potential predators (the red fox *Vulpes vulpes* and the grey wolf *Canis lupus*), and a potential competitor, the coypu *Myocastor coypus*. The beavers showed a mostly crepuscular behaviour. Although high temporal overlap was observed between the Eurasian beavers and the red foxes and grey wolves, the activity of the beavers did not overlap with that of the predators. Accordingly, the beavers were more active on the darkest nights, i.e., avoiding bright moonlight.

## 1. Introduction

“Ecosystem engineers” are species able to create or recreate, significantly modify, maintain, or destroy habitats (including microhabitats); thus, their behaviour may deeply influence organisms belonging to all trophic levels through interconnected processes [1,2,3,4]. As a rule, all animal communities are shaped by interspecific interactions, including competition and predation [5,6]. When in sympatry, competing species develop strategies to avoid each other by spatial, temporal, or diet segregation, as niche partitioning limits competitive interactions [7,8,9,10]. In detail, an interspecific overlap in temporal patterns of activity rhythms may imply interspecific competition, e.g., through direct interference (mostly amongst carnivores) and resource exploitation [11,12]. Assessing the temporal mechanisms of coexistence within animal communities may, thus, help determine variables shaping interspecific interactions, thereby improving our understanding of ecosystem processes [1,2,4]. The arrival of new species (both native and introduced) in an area may locally disrupt this equilibrium, forcing new behavioural adaptations in coexisting species [7,13]. In turn, the alteration of spatiotemporal patterns of activity can increase species’ exposure to local threats, including predation and interspecific competition [14,15,16].

The Eurasian beaver *Castor fiber* is the largest Eurasian rodent species [17]. It represents the most important “ecosystem engineer”, being able to deeply modify ecosystems to fulfill their ecological requirements, with significant linked hydrological, geomorphological, and ecological impacts [17,18]. Since 1920, reintroduction events and natural spread have triggered the recovery of the species in most of its original range, after being near extinct since Mediaeval times, up to a current minimum population estimate of about 1.5 million individuals worldwide [19]. Currently, the Eurasian beaver occurs with reproductive populations in most of its original range [19]. In Italy, the species persisted in the eastern Po valley up to the late 1500s [20,21]. Reintroduction programs occurred in the last century in neighbouring countries between the 1970s and the 1990s, promoting a recolonisation of many areas of these countries [19]. Since 2018, a single Eurasian beaver individual has been present in the Tarvisio area (NE Italy), probably due to natural dispersal from Austria [20]. In November 2020, another individual was camera-trapped in Val Pusteria (province of Bolzano), where two individuals might be currently present. Since 2019, the Eurasian beaver has also been present in Central Italy, with at least two populations in two river basins (Merse-Ombrone and Tevere rivers), where reproduction events have been confirmed for the first time since 2020 [22,23,24], proving their local establishment.

No information is available on the spatial and temporal behaviour of the beaver in Mediterranean ecosystems, and its temporal overlap with potential predators (i.e., with the grey wolf *Canis lupus*, and at least in terms of its kits with the red fox *Vulpes vulpes* [25,26,27]) and potential competitors (i.e., the coypu *Myocastor coypus*, a large rodent of South American origin that is very widespread in Europe [28]) is mostly unknown. Coypus and beavers share similar diets and habitats, including wooded rivers, lakes, small streams, and anthropised ecosystems [29]. In Central Italy, the coypu is the only semiaquatic meso-mammal species apart from the beaver. In Central-Northern Europe, beavers are mostly crepuscular or nocturnal, with some activity also in daylight possibly to limit encounters with predators and humans, avoiding bright moonlight nights [30,31,32,33]. Conversely, in the Mediterranean regions of Italy, all other considered species of this study show nocturnal behaviour [8,34,35,36,37], although the coypu may develop diurnal activity where predators and competitors are absent [34].

The main aim of this study was to assess the temporal mechanisms of interspecific coexistence in this area, where the Eurasian beaver reappeared after five centuries of local absence. In particular, we were interested in the spatiotemporal relationships between the beaver, i.e., the largest rodent species in our study area, and coexisting predators (i.e., the grey wolf and the red fox) and a potential competitor (i.e., the coypu). We predicted (*i*) that the temporal overlap between the beavers and their potential predators would be small, and (*ii*) that the beavers and coypus would show a low temporal overlap, with the smaller species avoiding the presence of the larger one.

## 2. Materials and Methods

### 2.1. Study Area

Our study was conducted in river basins, namely Ombrone-Merse (municipalities of Sovicille, Murlo, Monticiano, Buonconvento, Montalcino, and Civitella-Paganico) and Tevere (municipalities of Anghiari and Sansepolcro) in the Tuscany region (Central Italy), where the occurrence of at least 6–8 Eurasian beavers has been confirmed since at least 2019–2020 [22,23]. Deciduous riparian woodland is the main habitat type (87%), mostly represented by *Salix eleagnos* Scop., *Populus nigra* L., *Populus alba* L., *Alnus glutinosa* L. (Gaertn.), *Fraxinus ornus* L., and *Cornus mas* L. The alien species *Robinia pseudocacia* L. and *Acer negundo* L. also occur. The local climate is typically humid, with most precipitation occurring during autumn and winter; the mean annual precipitation rate is around 1500 mm. At least 16 wild mammal species (excluding bats) occur in this area [24]. The ungulates include the wild boar *Sus scrofa*, the roe deer *Capreolus capreolus*, and the alien fallow deer *Dama dama*. Apart from the Eurasian beaver, the large rodents included the crested porcupine *Hystrix cristata* and the coypu, although their local densities are not known. The grey wolf is the only large predator in the study area, with at least one pack of 4–6 individuals in the Ombrone-Merse study area and with the same number of individuals in the Tevere river basin (https://www.isprambiente.gov.it/it/attivita/biodiversita/monitoraggio-nazionale-del-lupo; accessed on 26 July 2022). The mesocarnivores (i.e., carnivores weighting less than 15 kg when adults [38]) include the red fox, which is the most abundant carnivore species in the study area, the wild cat *Felis silvestris*, the European badger *Meles meles*, the stone marten *Martes foina*, the pine marten *Martes martes* and the least weasel *Mustela nivalis*. The small mammal community is composed of wood mice *Apodemus* spp., bank voles *Chlethrionomys glareolus*, Italian water voles *Arvicola italicus*, and black rats *Rattus rattus*. Regarding domestic species, some domestic cats *Felis catus* and domestic sheep *Ovis aries* occur.

### 2.2. Camera Trapping

**Figure 1:**
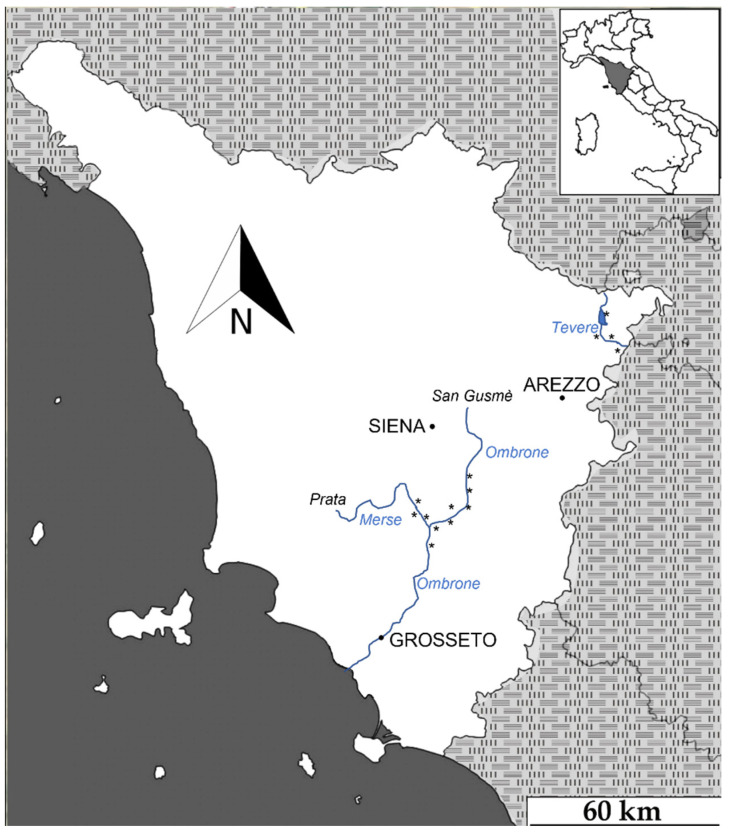
Position of camera trap stations in Tuscany (Central Italy). Asterisks represent camera-trap sites.

Data collection was conducted between March 2021 and May 2022. Camera traps (Browning SpecOps ©, *N* = 8) were placed at 14 stations (Figure 1), i.e., fixed georeferenced locations where each camera trap was tied with ropes and chains to trees or rocks. The camera trap stations were opportunistically selected by following the most recent signs of beaver presence (i.e., dams and lodges), all outside the protected areas. Apart from 2 camera traps placed at the dam, all of the others were placed in front of known burrows or lodges to capture the first emergence of individuals.

The stations were separated from each other by at least 800–1000 m to limit the risk of pseudo-replication [39]. The cameras were placed at heights of ~50–80 cm from ground level. They were activated 24 h/day, to take 1 video (30 s)/event; the cameras were checked once every 10 days to download data and replace batteries. The camera traps were rotated between stations once every ~100 nights. Camera trapping at each station lasted for 29–47 nights/season. The shortest monitoring time at one station was due to a beaver, which cut down the tree where the camera trap was placed. No significant temporal bias occurred in the sampling of stations in different seasons (Rayleigh test, Z = 12.47, *p* = 0.08 [39]). All the collected data were inserted into a dataset including the “species”, “number of individuals”, “date”, “hour”, “day phase” (i.e., diurnal or nocturnal), “station”, and “season” (i.e., cold months: October–March; warm months: April–September [36]). The night was defined as the period included between 1 h after sunset (i.e., at about 18:30 in winter and at about 22:00 in summer) and 1 h before sunrise (i.e., at about 07:00 in winter and at 05:00 in summer) [40,41].

### 2.3. Assessment and Overlaps of Activity Rhythms

Patterns of interspecific overlap of temporal activity patterns were estimated through the package “overlap” [42] in the software program R (version 3.6.1, R Foundation for Statistical Computing, Wien, Austria: www.cran.r-project.org (accessed on 5 July 2022 [43]). Analyses were carried out at the annual and, whenever possible, seasonal scales (i.e., cold months and warm months). Before the analysis, we removed from our dataset videos of the same species at the same site that occurred within less than 30 min (apart from cases where records belonged to clearly different individuals, e.g., an adult and a juvenile) to reduce pseudo-replication biases [42]. Thus, we considered as an “independent record” each video of a species remaining in the dataset after this filter was applied. We excluded from analyses ungulates, small mammals, and carnivores with less than 30 records (i.e., wild cat, domestic cat, Eurasian badger, stone marten, pine marten and least weasel [44]). Predation on beaver kits by Eurasian badgers and pine martens has been reported in the scientific literature; however, our camera trap data were too few to determine a temporal overlap with the rodents. Regarding the coypu, we also estimated the coefficient of overlap of temporal activity rhythms with the same species in the same study area (i.e., Merse river valley, Municipality of Sovicille, Siena) before the reappearance of the Eurasian beaver (i.e., in 2018).

We estimated the coefficient of overlapping (Δ) between the temporal activity patterns of beavers and all the other considered species (beaver–wolf, beaver–fox, beaver–coypu). The coefficients of overlapping ranged between 0 and 1 [42,45]. We calculated the Δ4 estimator when even the smallest sample of each pairwise comparison was >75 videos; we used the Δ1 estimator when the number of videos was <75 for at least one species of the pair [42,45]. Then, we estimated the 95% confidence intervals (hereafter, 95% CI) of the coefficient estimator based on 10,000 bootstrap replicates [46]. The temporal overlap was considered high if Δ > 0.75, intermediate if 0.50 < Δ < 0.75, and low if Δ < 0.50 [47]. Bootstrap tests were used to obtain a probability test that two sets of circular observations belonged to the same distribution, for all species pairs, with the function *compareCkern()* of the R-package “activity” [48]. The Hermans–Rasson test (r test) was used to estimate whether a random activity pattern was exhibited by the Eurasian beaver round-the-clock [49]. It was computed through the R package “CircMLE” [50]. The chi-square test was used to assess whether videos of each species were uniformly distributed throughout four moon phases, assessed through dedicated websites [36] and classified as follows: phase (1) from a new moon to 1/4 (epact days: 0–3; 26–29); phase (2) from 1/4 to 1/2 (epact days: 4–6; 21–25); phase (3) from 1/2 to 3/4 (epact days: 7–9; 17–20); phase (4) over 3/4 (epact days: 10–16).

## 3. Results

**Figure 2:**
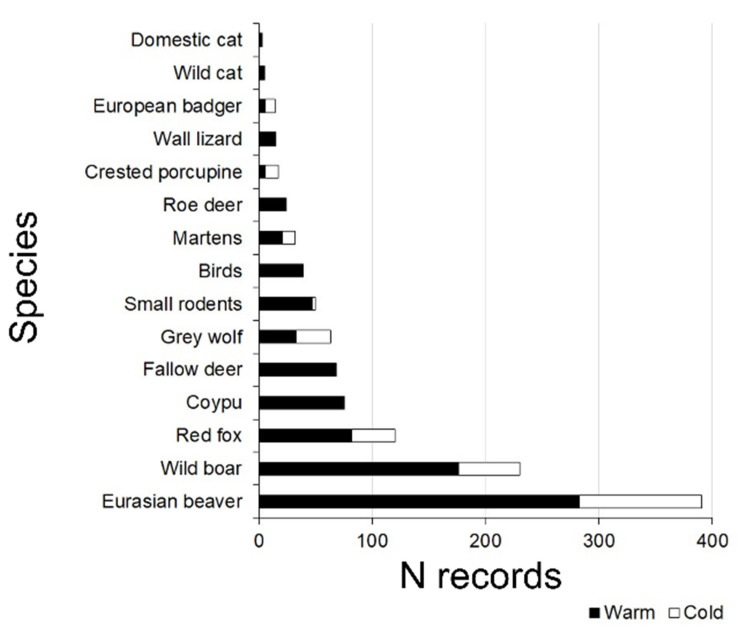
Number of independent video events per species and per season (cold and warm months; cf. Materials and Methods). Birds included 14 species: *Anas platyrhynchos*, *Spatula clypeata*, *Gallinula chloropus*, *Fulica atra*, *Egretta garzetta*, *Ardea cinerea*, *Casmerodius albus*, *Botaurus stellaris*, *Phasianus colchicus*, *Streptopelia turtur, Erithacus rubecula*, *Turdus merula*, *Turdus philomelos*, *Corvus cornix*, and *Garrulus glandarius*. Small mammals included 4 species: *Apodemus* spp., *Chletrionomys glareolus*, *Arvicola italicus*, and *Rattus rattus*. The least weasel is not included in this diagram, as it was camera-trapped only once.

We collected 1323 independent video events on 2765 camera trap days (i.e., number of camera traps × number of days of the camera trap survey). Beavers were recorded at all 14 stations. The videos depicted at least 32 species, including 14 birds (Figure 2).

**Figure 3:**
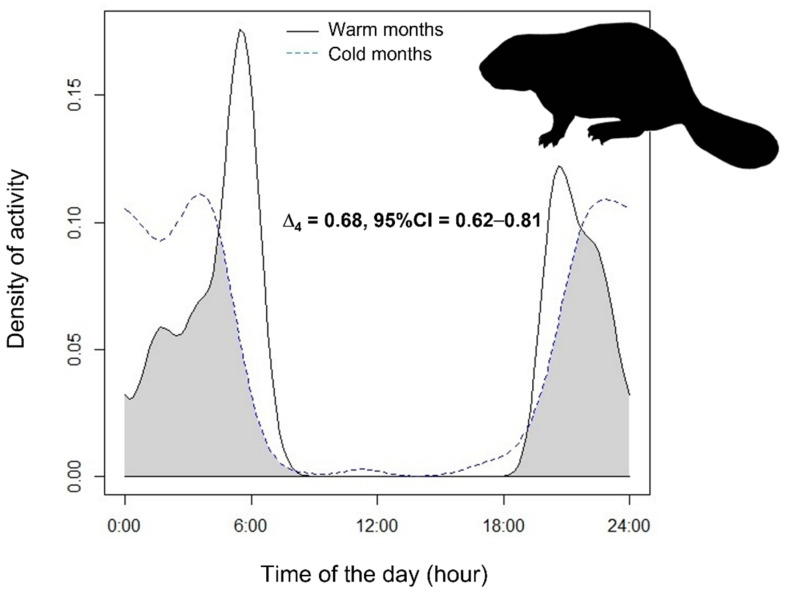
Interseasonal overlap of activity patterns expressed as kernel density estimates (coefficient Δ_4_) of the Eurasian beaver in Central Italy; 95% CI = 95% Confidence Intervals.

The Eurasian beaver was prevalently nocturnal, as its activity tended to stop before sunrise in 77% of cases, particularly in cold months. The activity peak occurred in the first part of the night in the cold months, and in the early morning in the warm months (Hermans–Rasson tests: r = 194.6–203.1; *p* < 0.001; Figure 3). We found an intermediate overlap in beaver activity rhythms across seasons (Figure 3). Throughout the year, the mean (±SD) duration of beaver activity, obtained by pooling all beaver videos from all stations in the same season, was 8 h 12 min ± 1 h 20 min.

**Figure 4:**
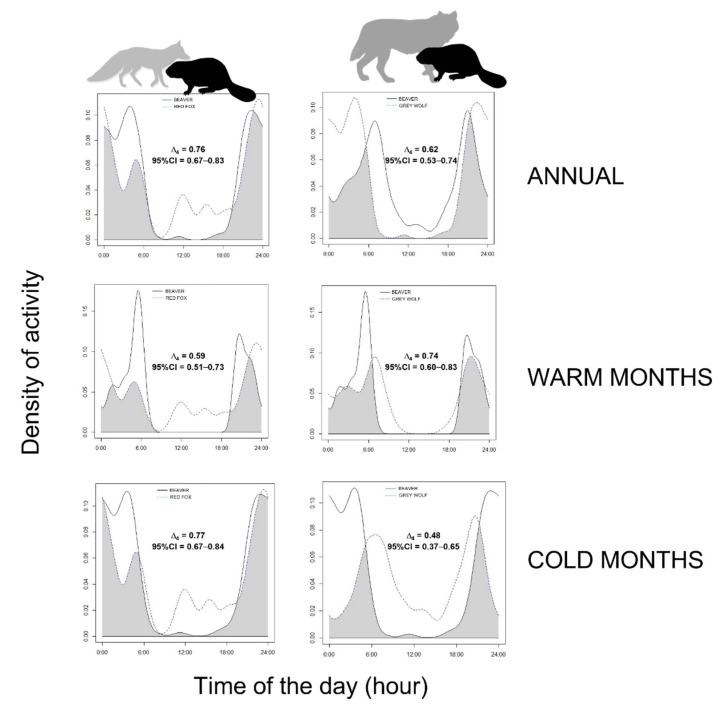
Interspecific overlap of activity patterns expressed as kernel density estimates (coefficient Δ_4_) between the Eurasian beaver and its main potential predators (**left**, red fox; **right**, grey wolf) in Central Italy; 95% CI = 95% Confidence Intervals.

During the course of the year and at the seasonal scale, we observed an intermediate to high overlap of activity rhythms between the Eurasian beaver and its main potential predators, apart from the wolf–beaver overlap in the cold months (Figure 4).

**Figure 5:**
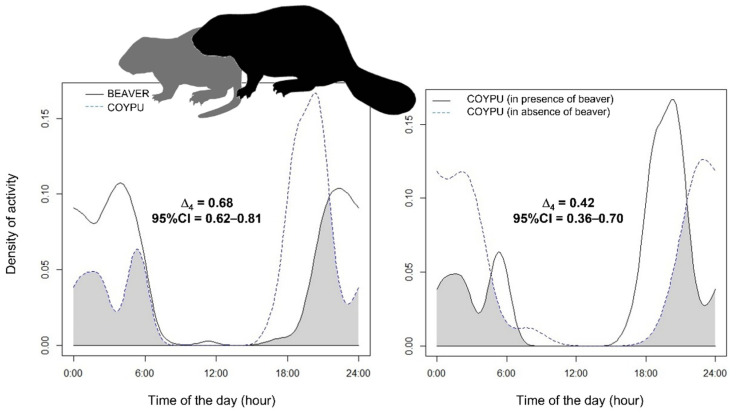
Interspecific overlap of activity patterns expressed as kernel density estimates (coefficient Δ_4_) between the Eurasian beaver and the coypu (on the **left**) in Central Italy. On the **right** is the overlap of activity rhythms between coypus in areas with and without beavers on the Merse river; 95% CI = 95% Confidence Intervals.

Regarding the coypu, it was possible to analyse its overlap of activity rhythms with the Eurasian beaver only at the annual level, given the negligible amount of coypu videos in the cold months (Figure 5). We found an intermediate overlap in activity patterns, with the coypus being more diurnal than beaver. However, we also observed a low overlap in patterns of activity rhythms between coypus in the same areas of the same river before and after the arrival of the beavers (Figure 5).

We observed a remarkable moonlight avoidance in the Eurasian beaver and coypu, which were mostly active on the darkest nights (Eurasian beaver: χ^2^ = 75.60, *p* << 0.001; coypu: χ^2^ = 17.24, *p* = 0.0006). Conversely, the grey wolf was mostly active on bright moonlight nights (χ^2^ = 14.25, *p* = 0.0003), and the activity of the red fox did not depend on moon phases (χ^2^ = 0.21, *p* = 0.97).

## 4. Discussion

In this work, we determined the patterns of activity rhythms of the Eurasian beaver in a Mediterranean ecosystem of recent reconquest [22,23,24]. The beaver was confirmed to be mostly a crepuscular–nocturnal species, as well as being present in the core of its European extent of occurrence [30,31,32,33]. In our study area, the beavers adjusted their activity rhythms following the (i) photoperiod (i.e., night length) and (ii) environmental temperatures, while not showing a high overlap of temporal activity between cold and warm months. Some irregular bouts of diurnal activity were recorded in cold months, possibly to limit activity during the coldest nights of the year. Conversely, the crepuscular and early-morning activity increased from winter to spring, while the night length decreased. Eurasian beavers may need to increase their activity to fulfill their nutritional requirements, as well as to thermoregulate, particularly in juvenile individuals (see [51,52] for the crested porcupine). In summer, when the nights are the shortest of the year, the peak in annual ambient temperature prevents the activity of this boreal rodent in daylight hours [51]. The Eurasian beaver may have evolved into the Russian steppes and adapted to live in cold climates [53], although its current wide range may force this species to withstand a wide environmental temperature range [19]. Its large and stocky body, short limbs, and abundant subcutaneous fat layer may represent adaptations to better cope with cold temperatures. Unlike most rodents of temperate areas, the beavers do not hibernate in our study area, and videos were collected throughout the entire year. However, the length of diel activity of the Eurasian beaver partly decreased (by at least one hour/night) in the colder months with respect to the warm months, and we collected a lower amount of beaver videos in October–March (*N* = 104 videos) compared to April–September (*N* = 283 videos). The limited food availability during winter drives the beavers to consume woody food sources along fixed river trails, mainly in the surroundings of their den [54]. Accordingly, over 73% of the videos in the cold months occurred at 1–15 m from the lodge, whereas in warm months only 48% of the videos were recorded near beaver dens, and most of them occurred in the surroundings of dams.

Throughout the year, the Eurasian beaver not only avoids diurnal hours, but also significantly reduces its activity on bright moonlight nights. This behaviour is adopted by some prey species [14,55,56,57] to limit encounters with their predators and with poachers. Where natural predators do not occur, the Eurasian beaver is mainly active on bright moonlight nights, allowing human avoidance, but with a foraging success improvement [33] since its vision is not well-adapted to movements in the dark. In our survey area, instead, Eurasian beavers coexist with grey wolves and red foxes. Beavers may represent an important food source for wolves [25,26,58], whereas the red foxes, badgers, and martens kill beaver kits only occasionally [27]. Despite an intermediate–high overlap in activity rhythms, the beavers may limit their encounters with wolves by being active on the darkest nights, when the predator is less active or less effective in hunting (cf. [59]). However, beavers have been living in the study area for only 3–4 years, and it is too early to discuss any relationships and mutual adaptations of prey and predator species. Thus, the spatiotemporal behaviour of the predators may have been shaped by that of their main prey occurring in the study area before the beaver’s occurrence.

Despite its wide and increasing distribution in Europe [28], interactions between the beaver and the invasive coypu have never been assessed before our research. The coypu is a large-sized semiaquatic rodent (8–10 kg of body weight) of South American origin, which was introduced to Europe for fur farms [28]. This species is currently listed amongst the invasive species of European concern, given the impacts it may exert on indigenous ecosystems. In most cases, coypus show nocturnal habits, although in the absence of predators and in urban or periurban areas, they may develop some diurnal behaviour [34]. In Italy, coypus are controlled in most of the Northern and Central regions, but the management plans are short on time and often not coordinated amongst different neighboring councils, thereby limiting their success [60,61]. Further data in the next years are needed to assess whether beavers and coypus are really competing species in our study area, also considering the difference in patterns of activity rhythms where both species are present and where each one is present without the other.

The Eurasian beaver and coypu have a very similar diet, although the first one is about 2–3 times larger (its weight can reach up 28–30 kg). In similar contexts, the larger species (whether native or not) usually displace smaller ones sharing similar niches as a sort of “wrecking ball effect”. For instance, the re-expanding native otter *Lutra lutra* in the United Kingdom are displacing the invasive American mink *Neovison vison*, reducing the local densities [62,63]. Similarly, increasing populations of the native Eurasian red squirrel *Sciurus vulgaris* in North-Eastern Italy have pushed out the populations of the invasive Siberian chipmunk *Eutamias sibiricus* outside the place where the smaller species has occurred for several decades, also resulting in a decline of its local density [64]. Where coypus were the only aquatic rodents in the Merse river, they showed nocturnal activity patterns, avoiding moonlight nights (cf. [34]). After the establishment of the Eurasian beaver, instead the coypus developed a more crepuscular–diurnal behaviour, although we have no evidence to exclude other hypotheses (i.e., differing from the beaver’s arrival) for this behaviour. For instance, coypus may have become diurnal to avoid wolves, their most important local predators [34,65].

## 5. Conclusions

Our study confirmed the effectiveness of camera trapping to determine seasonal and annual spatiotemporal relationships between semi-aquatic mammals, and between them and their potential predators.

With our work, we have provided the first evidence that also in Mediterranean habitats, characterised by higher temperatures as compared to temperate and boreal ones, the beavers have preserved a mostly crepuscular–nocturnal behaviour, with some diurnal bouts in spring, as in Central Europe, i.e., potential countries of origin of the beaver population currently occurring in Central Italy [22,23,24]. The beavers were mainly active on the darkest nights, i.e., reducing their activity on bright moonlight nights, when predators are mostly active both in our study area and in Central Europe (cf. [35,59]). Moreover, the partitioning of activity rhythms amongst similar species might be suggested to occur to avoid intraguild interference competition [66,67,68]. This theory has also been confirmed for large-sized rodents in riverine habitats. Further studies where beavers and coypus do not coexist are required to support this suggestion, i.e., in areas with beavers only and with coypus only. Intraguild interference competition plays a paramount role in interspecific interactions and may force coypus to be more active during daylight hours, making them easier to control. This is particularly important, as coypus are classified as invasive alien species by the EU Regulation 1143/2014, which requires prompt action for their removal [69].

## Data Availability

Not applicable.
